# Uncontrolled hypertension and associated factors among adult hypertensive patients in Ayder comprehensive specialized hospital, Tigray, Ethiopia, 2018

**DOI:** 10.1186/s12872-019-1091-6

**Published:** 2019-05-22

**Authors:** Gebrewahd Bezabh Gebremichael, Kalayou Kidanu Berhe, Teklewoini Mariye Zemichael

**Affiliations:** 1 School of Nursing College of Health Science, Mekelle Univesity, Tigray, Ethiopia; 2 Department of Nursing College of Health Science, Axum University, Tigray, Ethiopia

**Keywords:** Uncontrolled hypertension, Ayder comprehensive specialized hospital, Ethiopia

## Abstract

**Background:**

Despite the availability and improvement in diagnostic and therapeutic interventions with proven benefits in reducing cardiovascular morbidity and mortality, control rates of hypertension remain poor and grossly inadequate. Around one billion individuals are living with uncontrolled hypertension globally. Uncontrolled hypertension among hypertensive patients on treatment in Ethiopia ranges from 11.4 to 69.9%. Therefore, the aim of this study was to determine the magnitude and associated factors of uncontrolled hypertension among hypertensive patients in Ayder comprehensive specialized hospital, Tigray, Ethiopia 2018.

**Methods and materials:**

Hospital-based cross-sectional study design was conducted from February 16–April 30/2018. Simple random sampling method was used to select 320 participants. Data was collected using interviewer administered standard structured questionnaire. Self-care practice measuring tool was adopted from hypertension self-care activity level effects (H-scale). Data was entered to and cleaned by Epi Info version 7 and it was exported to SPSS version 22 for analysis. Binary logistic regression model (AOR, 95% CI and *p*-value < 0.05) was used to determine the predictors of uncontrolled hypertension.

**Result:**

From the total respondents, 164 (51.2%) were females. The mean age of the respondents was 53.83 + 14.52 years. Prevalence of uncontrolled hypertension was found 52.5%. Overweight (AOR = 4.527, 95% CI: 2.247–9.123), co-morbidity (AOR = 2.112, 95% CI: 1.218–3.662), non-adherence to anti-hypertensive medication (AOR = 2.062, 95% CI: 1.030–4.129), non-adherence to physical activity (AOR = 1.931, 95% CI: 1.074–3.470) and non-adherence to alcohol abstinence (AOR = 2.093, 95% CI: 1.109–3.948) are independent predictors of uncontrolled hypertension.

**Conclusion:**

the prevalence of uncontrolled hypertension is high. Patients’ adherence to antihypertensive medication, physical exercise and alcohol abstinence should be maximized. Weight reduction and early identification and management of co-morbidities are also crucial.

## Background

Uncontrolled hypertension is a major public health problem among hypertensive patients both in developed and developing countries [1–4]. Despite the availability and improvement in diagnostic options and therapeutic interventions of hypertension with proven benefits in reducing cardiovascular morbidity and mortality; BP control rates are grossly inadequate (< 30% controlled to < 140/90 mmHg) even among those diagnosed as hypertensive and taking anti-hypertension treatment [5, 6]. Around one billion individuals are living with uncontrolled hypertension globally [7].

Systolic Blood Pressure Intervention Trial (SPRINT) reported, intensive versus standard BP control (systolic BP of < 120 vs. < 140 mmHg) in adults with hypertension results in 25% risk reduction in major cardiovascular events and 27% reduction in all-cause mortality [8]. However, when hypertension remains uncontrolled, risks for long-term sequelae such as myocardial infarction, heart failure, stroke, and kidney disease significantly increase. For every 20 mmHg increase in systolic BP) to > 115 mmHg (or 10 mmHg increase in diastolic BP to > 75 mmHg), the risk of major cardiovascular and stroke events doubles [9]. Uncontrolled hypertension increases the risk of all-cause and cardiovascular disease mortality [10, 11]**.**

Majority of studies in Africa had shown that less than a third of patients achieve treatment goals [12]. A meta-analysis also showed that in most Sub-Saharan Africa (SSA), the control of BP to the target level (140/90) is less than 30% [13]. Few studies conducted in Ethiopia revealed that the prevalence of uncontrolled hypertension among patients on treatment varied from 11.4% in Gondar University Hospital to 59.9% in Tikur Anbessa Hospital and 69.9% in Zewditu Memorial Hospital [14–16].

Even though there is inconsistency among studies, multiple factors were found to contribute for uncontrolled hypertension. Non-adherence to anti-hypertensive therapy and dietary approach to stop hypertension (DASH diet), high salt intake, alcohol intake, smoking, physical inactivity and overweight/obesity are among the major contributing factors to uncontrolled hypertension [14, 17–19]. Other factors such as sex, age, disease duration and co-morbidities also have association with uncontrolled hypertension [17, 20].

One of the key Sustainable Development Goals adopted by the World Health Assembly in 2013 was to lower the prevalence of raised blood pressure by 25% by 2025 [21]. Improvement in the management and control of hypertension will require an understanding of the factors that affect blood pressure control. Although there are a few attempts the reasons for uncontrolled hypertension remain unclear in low income countries and have been insufficiently studied in Ethiopia. Thus, this study aimed to assess the prevalence and predictors of uncontrolled blood pressure in hypertensive patients attending in Ayder comprehensive specialized hospital, Tigray, Ethiopia.

## Methods

### Study area and period

ACSH is a university hospital in Mekelle, Tigray region commenced rendering all the specialized and non-specialized services including special clinic services [22]. The hospital provided follow-up care for 540 hypertensive patients according to data registered in 2017 prior to data collection period. The study was conducted from February 16 to April 30/2018.

### Study design and population

Hospital-based cross-sectional study design was conducted among adult hypertensive patients attending in Ayder comprehensive specialized hospital.

### Inclusion and exclusion criteria

All adult (≥ 18 years old) hypertensive patients who were on anti-hypertensive treatment and follow up for at least 6 months at time of data collection were included in this study. However unconscious and critical ill hypertensive patients and pregnant mothers were excluded.

### Sample size determination

Sample size was determined using single population proportion formula and considering the following assumptions: Prevalence (p) uncontrolled hypertension 0.7 [16], 푍 = standard normal distribution at 95% confidence level of 푍훼/2 = 1.96 and margin of error (푑) = 5%:

$$ \mathrm{N}=\frac{{\left({\mathrm{Z}}_{\upalpha /2}\right)}^2\ \mathrm{p}\ \left(1-\mathrm{p}\right)}{{\mathrm{d}}^2}=\frac{(1.96)^2\mathrm{X}\ 0.7\ \left(1-0.7\right)\ }{(0.05)^2}=320 $$

### Sampling procedure and techniques

A sampling frame was created using the patients’ medical registration number and participants were selected using simple random sampling technique from the sampling frame.

### Study variables

#### Independent variables

The independent variables were **socio-demographic variables (**age, sex, household monthly income in Ethiopian birr, marital status, religion, educational status, occupation, ethnicity and residence), **clinical characteristics of patients (**family history of hypertension, Body mass index, availability of BP cuff at home, BP monitoring at home or any else, presence of co-morbidity, duration of the disease and number and type of medication) and **behavioral practices** (adherence to anti-hypertensive medication physical activity, dietary management, moderation of alcohol and smoking status) of the participants.

#### Dependent variable

Uncontrolled hypertension was the dependent variable.

### Data collection tool

Data was collected using interviewer-administered structured questionnaire and document review. The questionnaire contains two parts. Part I contains socio-demographic characteristics, clinical profile and knowledge about hypertension and its management. Part II contains questions related to hypertension self-care practices such as medication adherence, dietary management (low salt diet and DASH diet), smoking status, physical activity, weight management and alcohol intake. The self-care practice measuring tool was adopted from hypertension self-care activity level effects (H-scale) [23].

### Operational definitions

#### Educational status

Is categorized in to four mutually exclusive categories. Can’t read and write includes participants who are not able to read and write. Can read and write includes participants who never took or followed formal education; however they might take informal education (e.g. religious education) thus they are able to read and write. Primary includes participants who followed primary level formal education. Secondary educational status includes participants who followed secondary level formal education. College and above includes participants who are honored of college diploma and above.

#### Uncontrolled hypertension

Is defined as systolic blood pressure ≥ 140 mmHg and/or diastolic blood pressure ≥ 90 mmHg in patients taking anti-hypertensive treatment [24].

### Scoring method

Hypertension Self-Care Activity Level Effects (H-SCALE) includes:

#### Medication adherence

Three items were used to assess the number of days in the last week that an individual: [1] takes blood pressure medication, [2] takes it at the same time every day and [3] takes the recommended dosage. Responses were summed (range, 0–21), and participants reported that they followed these 3 recommendations on 7 out of 7 days were considered adherent (score = 21).

#### Dietary management (Low-salt diet and DASH diet)

Twelve items were used to assess practices related to eating a healthy diet, avoiding salt while cooking and eating, and avoiding foods high in salt content. A mean score was calculated. A score of 6 or better (indicating that participants followed low-salt diet practices on 6 out of 7 days) was considered adherent.

#### Physical activity

Physical activity was assessed by 2 items. “How many of the past 7 days did you do at least 30 minutes total physical activity?” and “how many of the past 7 days did you do a specific exercise activity (such as swimming, walking or biking) other than what you do around the house or as part of your work?” Responses were summed (range, 0–14). Participants who scored ≥8 were coded as adhering to physical activity recommendations.

#### Smoking

Smoking status was assessed with 1 item, “How many of the past 7 days did you smoke a cigarette or cigar, even just one puff?” Respondents who reported 0 days were considered a nonsmoker. All others were categorized as smokers.

#### Weight management

Ten items were used to assess activities undertaken in the past 30 days to manage weight through dietary practices and physical exercise. Response categories ranged from strongly disagree [1] to strongly agree [5]. Participants who agree or strongly agree with all 10 items (score ≥ 40) were considered to be following good weight management practices.

#### Alcohol

Adherence to JNC7 recommendations was deemed to be alcohol abstinent. Participants who reported not drinking any alcohol in the last 7 days or who indicated that they usually didn’t drink at all was considered abstainers. All others were non adherent [23].

### Knowledge about lifestyle management of hypertension

- **Good knowledge:** knowledge score above the mean value on hypertension evaluation of lifestyle and management (HELM) scale.
- **Poor knowledge**: knowledge score below the mean value on hypertension evaluation of lifestyle and management (HELM) scale.

Hypertension evaluation of lifestyle and management scale which included 14 items was used to assess respondents’ knowledge [25]. The numbers of questions are modified to 10 as questions “7 and 8” are country specific and questions “12 and 13” does not meet the study objectives. The tool contains selected response items with the right answer coded as “1” and wrong answer as “0”.

### Data quality assurance

Data collectors and supervisors were trained for 1 day on the data collection approach of the study. The questionnaire was translated to Tigrigna language and back translated into English by another person to check for consistency and meaning and data was collected using the Tigrigna version questionnaire. A Pretest was conducted in 5% of the sample size in Axum comprehensive specialized hospital to see the applicability of the instruments and was ratified accordingly. Continuous follow-up and supervision was made by the supervisor and principal investigator throughout the data collection period. Collected data was reviewed and checked daily for completeness and consistency at the spot during data collection time.

### Data processing and analysis

Data was checked, cleaned and entered into Epi Info version 7 and then it was exported to SPSS version 22.0 for analysis. Descriptive statistics including frequencies, percentages, ranges, mean and standard deviations were done and presented using tables, figures and texts. Binary logistic regression was done to identify factors which were associated with the self-care practice. Variables which were found to have an association with the outcome variable at *P*-value < 0.25 in the bivariate regression analysis were entered to the multivariable logistic regression model. The magnitude of association between independent and dependent variables was measured using odds ratios and 95% confidence interval (CI) with significant level (*P*-value < 0.05).

### Ethical consideration

Ethical clearance was received from Mekelle University Collage of Health Science research committee. Informed consent was obtained and participation was fully based on the willingness of participants. The respondents were allowed to refuse or discontinue participation at any time. Information was recorded anonymously. Confidentiality and privacy were ensured throughout the study.

## Result

### Socio-demographic characteristics

A total of 320 adult hypertensive respondents were interviewed using a structured questionnaire and all questionnaires were included in the analysis which makes 100% of response rate. More than half of the total respondents (51.2%) were females. The mean age of the respondents was 53.83 + 14.52 years with a minimum age of 19 years old and maximum age of 85 years old. Majority of the respondents 227 (70.9%) were less than 65 years old and 230 (71.9%) of respondents were married. Most of the respondents 253 (79.1%) were orthodox Christian followers and 288 (90%) respondents were Tigrawot in ethnicity. One hundred thirty-eight (43.1%) of the respondents were not able to read and write and 86 (26.9%) of the respondents were self-employed. Majority of the study participants 177 (55.3%) had low monthly income and 232 (72.5%) of the respondents were urban residents (See Table 1).

**Table 1:** Sociodemographic characteristics hypertensive patients attending at ACSH, Mekelle, Tigray Region, Ethiopia 2018 (*n* = 320)

Variables	Category	Controlled BP	Uncontrolled BP	Total
Sex	Male	64 (20.0)	92 (28.8)	156 (48.8)
	Female	88 (27.5)	76 (23.8)	164 (51.2)
Age	< 65	118 (36.9)	108 (33.8)	226(70.6)
	≥ 65	34 (10.6)	60 (18.8)	94 (29.4)
Marital status	Married	123 (38.4)	119 (37.2)	242 (75.6)
	Single	14 (4.4)	18 (5.6)	32 (10.0)
	Divorced	5 (1.6)	11 (3.4)	16 (5.0)
	Widowed	10 (3.1)	20 (6.2)	30 (9.4)
Educational status	Can’t read & write	60 (18.8)	77 (24.1)	137 (42.8)
	Can read & write	22 (6.9)	32 (10)	54 (16.9)
	Primary	12 (3.8)	10 (3.1)	22 (6.9)
	Secondary	10 (3.1)	8 (2.5)	18 (5.6)
	College and above	48 (15)	41(12.8%	89 (27.8)
Income (Ethiopian birr)	< 500	10 (3.1)	12 (3.8)	22 (6.9)
	500–1000	55 (17.2)	66 (20.6)	121(37.8)
	> 1000	87 (27.2)	90 (28.1)	177(55.3)
Residence	Urban	112 (35.0)	120 (37.5)	232 (72.5)
	Rural	40 (12.5)	48 (15.0)	88 (27.5)

### Knowledge

Mean score of knowledge of respondents was 4.3 + 1.19 with a minimum score of 1 and a maximum score of 8 (See Table 2).

**Table 2:** Knowledge distribution of hypertensive patients attending at ACSH, Mekelle, Tigray Region, Ethiopia, 2018 (*n* = 320)

Variable	characteristics	Controlled BP	Uncontrolled BP	Total
Knowledge	Good	81 (25.3)	65 (20.3)	146 (45.6)
	Poor	71 (22.2)	103 (32.2)	174 (54.4)

### Health profile related factors

Ninety-seven (30.3%) of the total respondents had a family history of hypertension and only 13 (4.1%) of the respondents had BP cuff at home. The mean duration of hypertension was 3.50 + 3.07 with a minimum of 0.5 year and a maximum of 20 years. A significant number of respondents,215 (67.2%) had normal BMI. One hundred sixty-two of the respondents(50.3%) had medically confirmed co-morbidity. One hundred thirty-seven (42.8%) of the respondents checked their BP regularly at least twice a month at home, health institution or any else. Ninety-six respondents (30%) had ever missed follow-ups. Majority of the respondents, 228 (71.2%) took less than or equal to two types of antihypertensive medication (See Table 3).

**Table 3:** Distribution of health profile of hypertensive patients attending at ACSH, Mekelle, Tigray Region, Ethiopia 2018 (*n* = 320)

Variables	Characteristics	Controlled BP	Uncontrolled BP	Total
Family history	Yes	45 (14.1)	52 (16.2)	97 (30.3)
	No	107 (33.4	116 (26.2)	223 (69.7)
Duration of disease	< 2	33 (10.3)	41(12.8)	74 (23.1)
	2–3.9	72 (22.5)	63 (19.7)	135 (42.2)
	≥ 4	47 (14.7)	64 (20)	111 (34.7)
BMI	18.5–24.9	116 (36.2)	99 (30.9)	215 (67.2)
	25–29.9	18 (5.6)	64 (20)	82 (25.6)
	> 30	2 (0.6)	5 (1.6)	7 (2.2)
	< 18.5	16 (5)	0 (0)	16 (5%)
Co-morbidity	Yes	60 (18.8)	101 (31.6)	161(50.3)
	No	92 (28.8)	67 (20.9)	159 (49.7)
BP check	Yes	73 (22.8)	64 (20)	137 (42.8)
	No	79 (24.7)	104 (32.5)	183 (57.2)
Follow up miss	Yes	37 (11.6)	59 (18.4)	96 (30.0)
	No	115 (35.9)	109 (34.1)	224 (70)
No of type of anti-HTN medications	≤ 2	119 (37.2)	109 (34.1)	228 (71.2)
	≥ 3	33 (10.3)	59 (18.4)	92 (28.8)
Medication adherence	Adherent	123 (38.4)	114 (35.6)	237 (74.1)
	Non-adherent	29 (9.1)	54 (16.9)	83 (25.9)
Adherence to dietary management	Adherent	106 (33.1)	96 (30)	202 (63.1)
	Non-adherent	46 (14.4	72 (22.5)	118 (36.9)
Adherence to physical exercise	Adherent	95 (29.7)	63 (19.7)	158 (49.4)
	Non-adherent	57 (17.8)	105 (32.8)	162 (50.6)
Nonsmoking adherence	Adherent	152 (47.5)	165 (51.6)	317 (99.1)
	Non-adherent	0 (0.0)	3 (0.9)	3 (0.9)
Adherence to Alcohol abstinence	Adherent	119 (37.2)	96 (30)	215 (67.2)
	Non-adherent	33 (10.3)	72 (22.5)	105 (32.8)
Adherence to Weight management	Adherent	73 (22.8)	57 9 (17.8)	130 (40.6)
	Non-adherent	79 (24.7)	111(34.7)	190 (59.4)

### Uncontrolled hypertension and associated factors

The magnitude of uncontrolled hypertension was found to 52.5% (95% CI, 47.2–58.1%). Fifteen variables were found significantly associated variables in the bivariate logistic regression. However, only five variables (BMI, co-morbidity, adherence to medication and physical activity and alcohol abstinence) were found statistically significant predictors of uncontrolled hypertension at *p*-value < 0.05 in the Multivariable logistic regression model.

Overweight patients were found 4.527 more likely to have uncontrolled hypertension compared to those with normal weight (AOR = 4.527, 95% CI: 2.247–9.123). Hypertensive patients with co-morbidity had 2.112 more risk of uncontrolled hypertension than their counterparts (AOR = 2.112, 95% CI: 1.218–3.662). Hypertensive patients who were non-adherent to anti-hypertensive medication had 2.062 more risk of uncontrolled hypertension (AOR = 2.062, 95% CI: 1.030–4.129). Non-adherence to physical activity had1.931more risk of uncontrolled hypertension (AOR = 1.931, 95% CI: 1.074–3.470). Similarly, non-adherence to alcohol abstinence had 2.093 more risk of uncontrolled hypertension (AOR = 2.093, 95% CI: 1.109–3.948) (See Table 4).

**Table 4:** Bivariate and multivariable logistic regression analysis result for significant variables among hypertensive attending at ACSH, Mekelle, Tigray Region, Ethiopia 2018 (*n* = 320)

Variable	Category	Controlled	Uncontrolled	COR	AOR	P-value
Sex	Male	64 (20.0)	92 (28.8)	1.664 (1.069–2.591)	1.167 (0.662–2.056)	0.593
	female	88 (27.5)	76 (23.8)	1		
Age	< 65	118 (36.9)	108 (33.8)	1	1	
	≥ 65	34 (10.6)	60 (18.8)	1.928 (1.175–3.163)	1.212 (0.600–2.446)	0.593
Educational status	Can’t read & write	60 (18.8)	77 (24.1)	1.502 (0.879–2.569)	.659 (0.302–1.435)	0.293
	Can read & write	22 (6.9)	32 (10)	1.703 (0.859–3.376)	0.648 (0.273–1.536)	0.324
	Primary	12 (3.8)	10 (3.1)	0.976 (0.382–2.490)	0.560 (0.183–1.719)	0.311
	Secondary	10 (3.1)	8 (2.5)	0.937 (0.338–2.594)	0.655 (0.207–2.070)	0.471
	College and above	48 (15)	41(12.8%	1	1	
Duration of hypertension	< 2 years	33 (10.3)	41(12.8)	0.912 (0.504–1.651)	1.139 (0.544–2.385)	0.730
	2–4 years	72 (22.5)	63 (19.7)	0.643 (0.387–1.066)	0.910 (0.489–1.696)	0.767
	≥ 4 years	47 (14.7)	64 (20)	1	1	
BMI	18.5–24.9	116 (36.2)	99 (30.9)	1	1	1
	25–29.9	18 (5.6)	64 (20)	4.166 (2.315–7.498)	4.527 (2.247–9.123) *	0.000
	≥ 30	2 (0.6)	5 (1.6)	2.929 (0.556–15.431)	3.711(0.618–22.283)	0.152
	< 18.5	16 (5)	0 (0)	.000(.000 …)	.000 (.000 …)	0.998
Co-morbidity	Yes	60 (18.8)	101 (31.6)	2.311(1.476–3.620)	2.112 (1.218–3.662) *	0.008
	No	92 (28.8)	67 (20.9)	1	1	
BP-check up	Yes	73 (22.8)	64 (20)	1	1	
	No	79 (24.7)	104 (32.5)	1.502 (0.962–2.344)	1.478 (0.830–2.630)	0.184
Follow up miss	Yes	37 (11.6)	59 (18.4)	1.682 (1.033–2.739)	1.225 (0.658–2.279)	0.522
	No	115 (35.9)	109 (34.1)	1	1	
Number of types of medications	≤ 2	119 (37.2)	109 (34.1)	0.512 (0.311–.844)	.671 (0.363–1.242)	0.204
	≥ 3	33 (10.3)	59 (18.4)	1	1	
Knowledge status	Good	81 (25.3)	65 (20.3)	1	1	
	Poor	71 (22.2)	103 (32.2)	1.808 (1.159–2.821)	.920 (0.512–1.655)	0.781
Medication adherence	Yes	123 (38.4)	114 (35.6)	1	1	
	No	29 (9.1)	54 (16.9)	2.009 (1.197–3.373)	2.062 (1.030–4.129) *	0.041
Adherence to dietary management	Yes	106 (33.1)	96 (30)	1	1	
	No	46 (14.4	72 (22.5)	1.728 (1.089–2.742)	0.949 (0.508–1.772)	0.869
Adherence to physical activity	Yes	95 (29.7)	63 (19.7)	1	1	
	No	57 (17.8)	105 (32.8)	2.778 (1.766–4.370)	1.931 (1.074–3.470) *	0.028
Adherence to alcohol abstinence	Yes	119 (37.2)	96 (30)	1	1	
	No	33 (10.3)	72 (22.5)	2.705 (1.654–4.423)	2.093 (1.109–3.948) *	0.023
Adherence to weight management	Yes	73 (22.8)	57 9 (17.8)	1	1	
	No	79 (24.7)	111(34.7)	1.799 (1.146–2.824)	1.508 (0.793–2.869)	0.210

## Discussion

The magnitude of uncontrolled hypertension was found high, 52.5% (95% CI: 47.2–58.1%). The rate of uncontrolled hypertension is in line with the study findings done in Thailand (53.4%) [26], Kwazulu-Natal (51%) [27], South Asia (58.0%) [28], Ghana (57.7%) [29], Nigeria 53.6% [30] and Jimma university hospital, Ethiopia (52.7%) [19]. However, this is higher than the findings reported from Israel (35.9%) and Gondar university hospital, Ethiopia (37%) [31, 32]. This could be explained by the lower rates of medication adherence and a slightly higher magnitude of co-morbidity in our study compared to the study done in Gondar, Ethiopia. The difference in the operational definition of uncontrolled hypertension among the studies could also be responsible for the discrepancy of the study results [32]. In addition, this might be due to sociocultural and behavioral differences of the population and healthcare services differences of the study settings.

In contrary, the magnitude of uncontrolled hypertension in this is lower than the study done in Panama (66.7%) [33], Western India 63.6% [34], Southern China (55.4%) [35], Morocco 82.8% [36], Kinshasa, Democratic Republic of the Congo (77.5%) [37], South Africa (75.5%) [38], Zimbabwe (67.2%) [39], Cameron (63.2%) [40], Ethiopia (63%) [41] and Zewditu Memorial Hospital, Ethiopia (69.9%) [16]. This inconsistency could be due to the difference in the; anti-hypertensive medication adherence rate, proportion of overweight and obesity, magnitude of co-morbidity, operational definition of hypertension, adherence to alcohol abstinence and age of study participants.

Compared to the other studies, our study reported a higher rate of medication adherence [33, 34, 37, 38, 41]. The other studies reported a higher prevalence of overweight and obesity [33, 35, 36, 39, 40]. Our study also had a low prevalence of co-morbidity among hypertensive patients. Most of the studies had high proportion co-morbidities or exclusively done among hypertensive patients with chronic co-morbidities [33, 36, 38–40]. The study done in democratic republic Congo has operationally defined uncontrolled hypertension as BP of ≥130/80 for hypertensive patients with chronic co-morbidities [37]. So that, this lower cut point could contribute to the increased prevalence of uncontrolled hypertension. Our study revealed higher adherence to alcohol abstinence compared to other study done in Ethiopia [41]. Compared to our study the studies done in China, Morocco, Democratic Republic of Congo, South Africa and Zimbabwe had higher proportion of older adults and even some of them were done exclusively among older adults [35–39]. Even though our study didn’t show significant association of age with uncontrolled hypertension, many previous studies revealed advanced age is an independent predictor of uncontrolled hypertension [19, 36, 41, 42]. Aging causes hypertension referred to as isolated systolic hypertension which is found primarily in elderly people by stiffening of the aorta [43].

Overweight was found significant predictor of uncontrolled hypertension. This is supported by the studies done in China and Zimbabwe [35, 39]. Other study done in Jimma university hospital, Ethiopia also revealed overweight and obesity were independent predictors of uncontrolled hypertension [19]. Higher BMI (overweight and obesity) is one major contributing factor for hypertension and many health studies have consistently identified that BMI and blood pressure have a direct and apparent dose-response relationship [44]. The mechanism by which obesity directly causes hypertension is under investigation. Activation of the sympathetic nervous system, the amount of intra-abdominal and intra-vascular fat, sodium retention leading to increase in renal reabsorption, and the renin-angiotensin system are considered to have important functions in the pathogenesis of obesity related hypertension [45].

Co-morbidity had a significant association with uncontrolled hypertension. This is consistent with the studies done in South Asia and China, which showed diabetic and kidney disease co-morbidities were associated with uncontrolled hypertension [28, 35]. This is also in line with the study done in Gondar university hospital, Ethiopia which showed co-morbid hypertensive patients were more likely to have uncontrolled hypertension [32]. Many chronic diseases are secondary causes of hypertension so that controlling hypertension among hypertensive patients with other chronic co-morbidities might be challengeable.

Non-adherence to anti-hypertensive medication was an independent predictor of uncontrolled hypertension. This is in line with studies done in and Southern California [46], South Asia [28] and Zimbabwe [39] and Cameron [40] in which good adherence to antihypertensive medication was found protective to uncontrolled hypertension. It is also supported by the study done in Ghana and University of Gondar hospital, Ethiopia which showed poor adherence or non-adherence to anti-hypertensive medication was found statistically associated with uncontrolled hypertension [29, 32]. This congruency could be due to the good adherence to antihypertensive medication is essential to control hypertension and reduces blood pressure. Anti-hypertensive medications lower and control high blood pressure by increasing vasodilatation and decreasing vasoconstriction, increasing urine output and blocking the sympathetic activation of heart [47].

Non-adherence to physical activity was associated with uncontrolled hypertension. This is similar with the study done in China which showed that lack of physical activity was statistically associated with uncontrolled hypertension [35]. Epidemiological studies have evidenced that physical activity results in significant BP and weight reduction. Sedentary life, which is a known predictor of obesity, is one of the major risk factors of high blood pressure and thus non-adherence to physical exercise makes difficult to control hypertension [44]. Although precise mechanisms have yet to be fully elucidated, available data have provided enough information to establish biologically plausible mechanisms for the relationship between physical activity and hypertension. Physical exercise may prevent increases in BP through beneficial alterations in insulin sensitivity, and autonomic nervous system function and vasoconstriction regulation. It also decreases high blood pressure by decreasing body weight and increasing renal function [48].

Non-adherence to alcohol abstinence was found significantly associated with uncontrolled hypertension. This is consistent with studies done in South Africa [38], Zimbabwe [39] and Jimma university teaching hospital, Ethiopia [19]. Health studies evidenced that Alcohol is one of the risk factors and is accountable for significant population burden of hypertension. Non-adherence of hypertensive patients to recommendations of alcohol intake makes more difficult to control hypertension [44]. Stimulation of the endothelium to release vasoconstrictors and loss of relaxation due to inflammation and oxidative injury of the endothelium by angiotensin II leading to inhibition of endothelium-dependent nitric oxide production is the major contributors of the alcohol-induced hypertension [49].

## Limitations

There might be recall bias and social desirability bias since the behavioral practice of the study participants were based on self-reports and performance of these behaviors was not observed and could not be confirmed. Since our study design is a cross-sectional study it doesn’t show temporal relationship. Hence, it is also difficult to confirm the cause and effect between the dependent and predictor variables. Besides the study was conducted in a relatively small sample size which may have an effect on generalizability.

## Conclusion

The magnitude of uncontrolled hypertension was found high. Non-adherence to anti-hypertensive medications, overweight, co-morbidity, non-adherence to physical exercise and non-adherence to alcohol abstinence were the independent predictors of uncontrolled hypertension. So that health care professionals and other stakeholders should promote overweight hypertensive patients to reduce their weight and maximize patients’ adherence to antihypertensive therapy, physical exercise and alcohol abstinence. Early identification and management of co-morbidities among hypertensive patients is crucial to control hypertension.

## Abbreviations

ACSH: Ayder comprehensive specialized hospital
AOR: Adjusted odds ratio
BMI: Body max index
BP: Blood pressure
CI: Confidence interval
COR: Crude odds ratio
CVD: Cardiovascular disease
DASH: Dietary approaches to stop hypertension
DF: Degree of freedom
HELM: Hypertension evaluation of lifestyle and management
H-SCALE: Hypertension self-care activity level effect
HTN: Hypertension
JNC: Joint National Commission
NCD: Non-communicable disease
OR: Odds ratio
SPSS: Statistical package for social sciences
US: United state
WHO: World Health Organization
